# Laryngeal Edema Following Ingestion of Glyphosate Potassium Salt

**DOI:** 10.1002/ccr3.73671

**Published:** 2026-09-30

**Authors:** Tatsuya Fujihara, Kaoru Kirita, Eisuke Okamoto

**Affiliations:** ^1^ Division of General Medicine Masuda Red Cross Hospital Masuda Japan; ^2^ Department of Anesthesiology Shimane University Faculty of Medicine Izumo Japan

**Keywords:** glyphosate potassium salt, herbicide poisoning, laryngeal edema, upper airway obstruction

## Abstract

Although glyphosate potassium salt formulations are considered less systemically toxic, they can cause direct upper airway mucosal injury, leading to severe laryngeal edema. Hoarseness after ingestion should prompt early laryngeal evaluation for impending airway compromise.

## 1 Case Illustrated

A 93‐year‐old woman with a history of hypertension, heart failure, osteoporosis, and dementia was transported to the emergency department approximately 2 h after having intentionally ingested an estimated 250 mL of diluted Roundup Maxload in an attempted suicide.

On arrival, the patient presented with hoarseness, accompanied by the following vital signs: respiratory rate, 24 breaths/min; blood pressure, 117/64 mmHg; pulse rate, 73 beats/min; and body temperature, 35.8°C. The Glasgow Coma Scale score was E4V4M6. Bronchoscopy revealed edema of the arytenoid region and vocal folds, which prompted endotracheal intubation to secure the airway.

The patient was admitted to the intensive care unit, wherein she underwent continuous renal replacement therapy for toxin‐induced hyperkalemia. At 3 h after arrival, nasogastric tube insertion proved difficult, requiring endoscopic guidance for tube placement. The patient was found to have marked swelling of the glottis and epiglottis (Figure 1). By hospital day 5, the laryngeal edema had improved, and the patient was successfully extubated. One month after admission, the patient was transferred to a rehabilitation facility for voice and swallowing function recovery.

**FIGURE 1:**
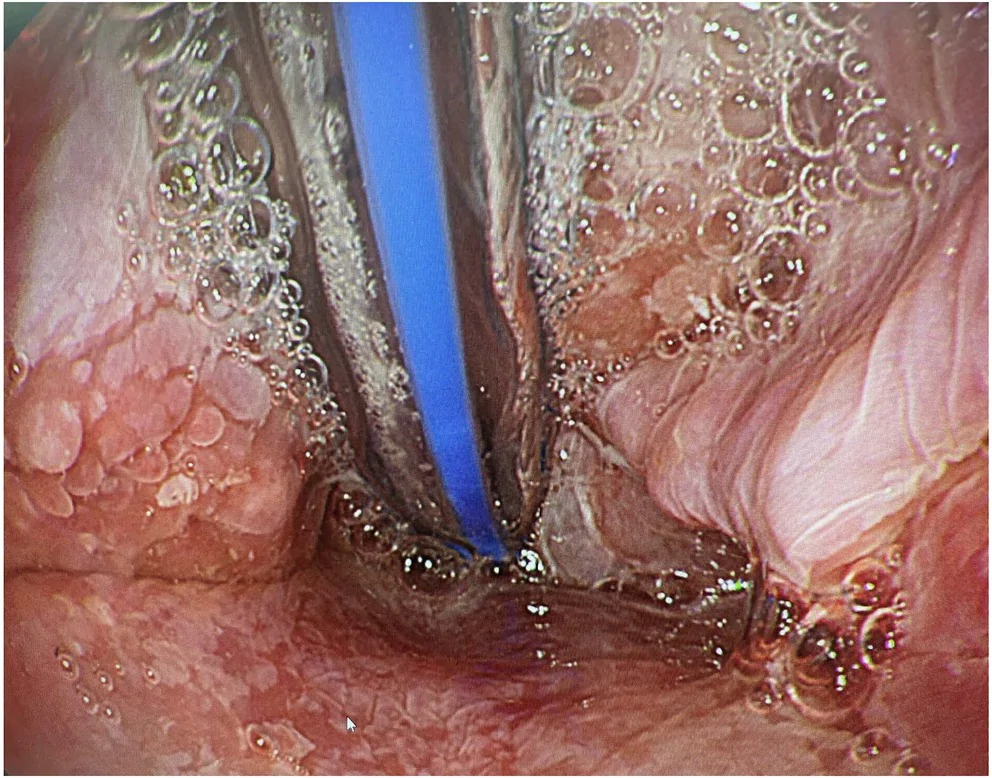
At 3 h after endotracheal intubation, endoscopy showed marked periglottic edema.

## 2 Discussion

Glyphosate‐surfactant herbicide products contain several types of surfactants, such as polyoxyethyleneamine, which can cause mucosal damage [1]. Although the surfactants used in glyphosate potassium salt formulations may pose a lower risk of injury to organs such as the lungs, liver, and kidneys [2], ingestion can still cause direct upper airway mucosal injury, leading to severe laryngeal edema. Hoarseness or dysphonia after ingestion should prompt early laryngeal evaluation because severe edema and airway compromise may develop rapidly.

## Author Contributions

**Tatsuya Fujihara:** visualization, writing – original draft. **Kaoru Kirita:** writing – review and editing. **Eisuke Okamoto:** writing – review and editing.

## Funding

The authors have nothing to report.

## Ethics Statement

The authors have nothing to report.

## Consent

Written informed consent for publication of this clinical image was obtained from the patient's relative.

## Conflicts of Interest

The authors declare no conflicts of interest.

## Data Availability

All relevant data supporting the findings of this study are included within the article.
